# Comparisons of prognostic value between brachial-ankle pulse wave velocity and estimated pulse wave velocity

**DOI:** 10.1038/s41440-026-02647-z

**Published:** 2026-05-08

**Authors:** Hack-Lyoung Kim, Haechan Cho, Hyun Sung Joh, Woo-Hyun Lim, Jae-Bin Seo, Sang-Hyun Kim

**Affiliations:** Division of Cardiology, Department of Internal Medicine, Seoul Metropolitan Government-Seoul National University Boramae Medical Center, Seoul National University College of, Medicine, Seoul, Republic of Korea

**Keywords:** Cardiovascular Diseases/epidemiology, Proportional Hazards Models, Pulse Wave Analysis, Risk Assessment, Vascular Stiffness.

## Abstract

Arterial stiffness is a key marker of vascular aging. We compared the prognostic performance of brachial-ankle pulse wave velocity (baPWV) and estimated PWV (ePWV) for major adverse cardiovascular events (MACE). We retrospectively analyzed adults aged 40-75 years who underwent baPWV at a tertiary center (n = 9521). ePWV was computed from age and mean blood pressure. The primary endpoint was MACE (cardiac death, non-fatal myocardial infarction, coronary revascularization, non-fatal ischemic stroke). During a median follow-up of 3.77 years, 271 MACEs occurred (2.8%). Multivariable Cox regression models showed that higher arterial stiffness by both baPWV and ePWV was independently associated with increased MACE risk (*P* < 0.05 for each). Consistently, baPWV identified stepwise increases in risk from lower to higher categories and produced larger effect estimates. However, ePWV retained independent prognostic value but with a weaker gradient. With dichotomized cutoffs, C-index values were similar for baPWV and ePWV (0.736 *vs*. 0.728; *P* = 0.323). When participants were stratified into tertiles, baPWV showed superior discrimination, yielding a higher C-index than ePWV (0.755 *vs*. 0.729; *P* = 0.033) and clearer separation of Kaplan-Meier curves across risk strata. These findings indicate that both measures add information beyond traditional risk factors, but baPWV provides stronger risk stratification, particularly when risk is partitioned into multiple levels. ePWV remains a practical alternative in settings where device-based testing is not feasible.

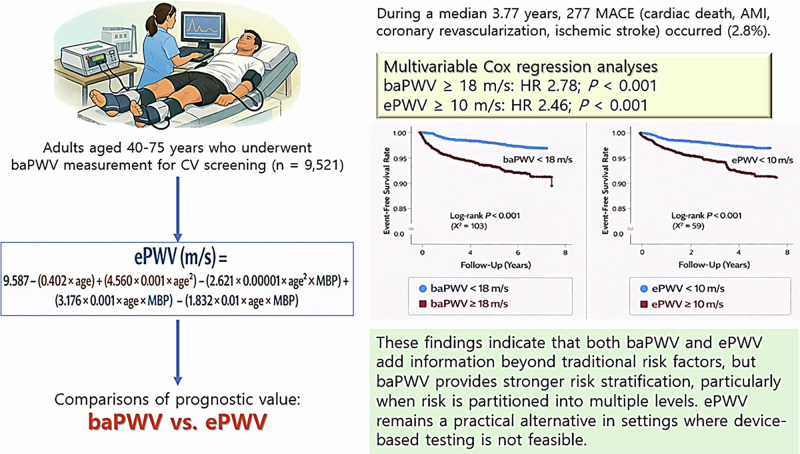

## Introduction

Arterial stiffness is a central marker of vascular aging and an independent predictor of cardiovascular morbidity and mortality [1]. Pulse wave velocity (PWV) is the most widely used and validated noninvasive measure of arterial stiffness, providing important prognostic information beyond traditional cardiovascular risk factors [2]. Among several modalities, brachial-ankle pulse wave velocity (baPWV) has been extensively applied in clinical practice, particularly in Asian countries, due to its simplicity, reproducibility, and feasibility in large-scale screenings [2]. Numerous studies have demonstrated the prognostic significance of baPWV for cardiovascular outcomes in hypertensive patients, patients with coronary artery disease, and general populations [3–5].

Despite its clinical utility, baPWV measurement requires dedicated equipment, which can limit its widespread adoption in routine practice. To overcome these limitations, estimated PWV (ePWV), a formula-based index derived from age and blood pressure, has been proposed [6, 7]. Because ePWV can be easily calculated from basic clinical information without specialized devices, it has attracted attention as a potentially useful tool for large-scale epidemiological studies and clinical risk stratification [8]. Importantly, ePWV also offers the advantage of accessibility for individuals in resource-limited settings, as it can provide valuable information on arterial stiffness without the costs associated with specialized testing. Recent evidence has suggested that ePWV also provides significant prognostic information, with performance comparable to directly measured PWV in certain populations [8, 9].

However, direct comparisons between baPWV and ePWV in terms of their prognostic value remain limited [10, 11]. Given that baPWV captures arterial stiffness via direct hemodynamic measurements and ePWV reflects estimated vascular aging from demographic and hemodynamic parameters, their predictive utility may differ across diverse patient populations. Clarifying the relative prognostic roles of baPWV and ePWV is therefore clinically important, particularly in patients undergoing cardiovascular evaluation.

The present study aims to directly compare the prognostic value of baPWV and ePWV for predicting major adverse cardiovascular events in a large cohort. By examining their relative performance, we seek to provide new insights into whether simple formula-based estimation can substitute for, or complement, direct hemodynamic measurement in clinical practice.

Point of view

- Clinical relevance
  baPWV provides superior risk stratification for cardiovascular events, while ePWV serves as a practical alternative when device-based measurement is not available.
- Future direction
  Prospective multicenter studies across diverse Asian populations are needed to validate standardized cutoffs and to determine whether PWV-guided management improves clinical outcomes.
- Consideration for the Asian population
  Differences in vascular aging, hypertension patterns, and healthcare accessibility across Asian populations support a dual strategy using both baPWV and ePWV for risk assessment.

## Methods

### Study population

**Fig. 1:**
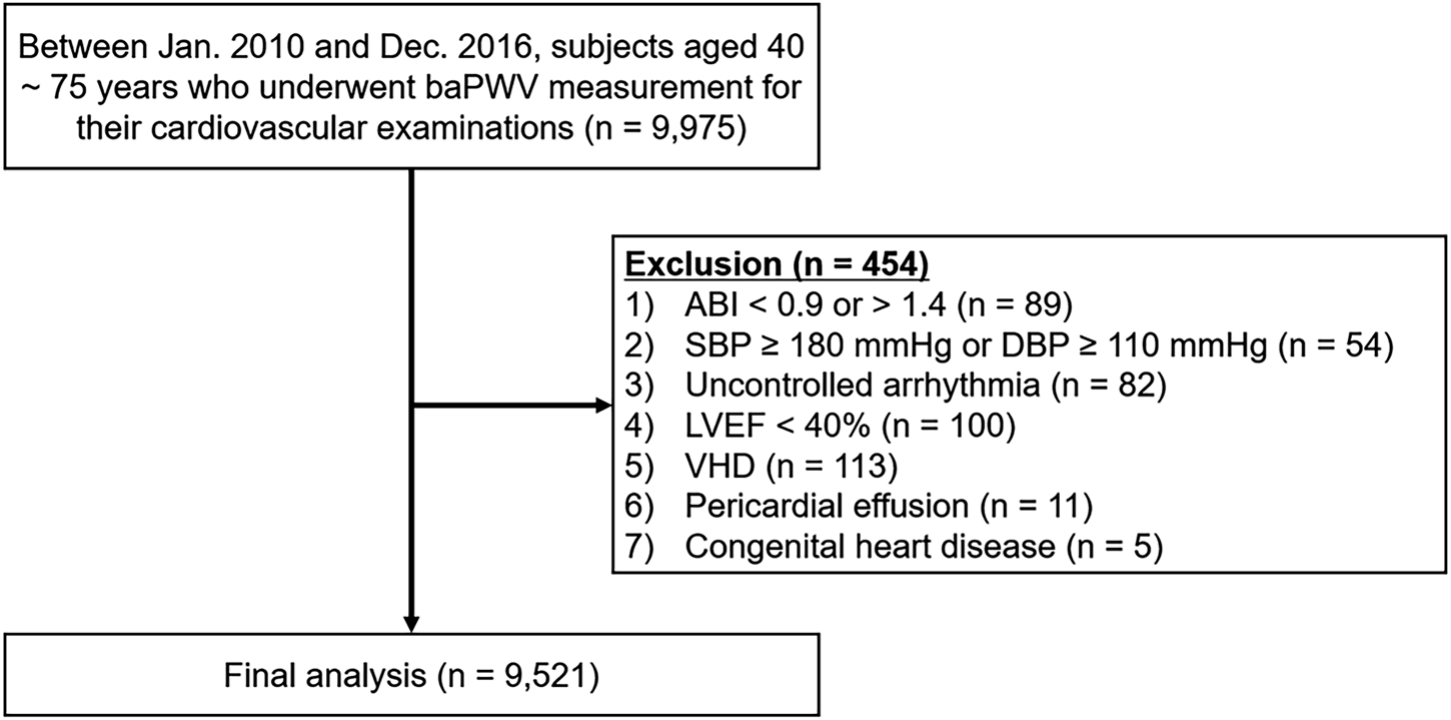
Flowchart of participant enrollment. ABI ankle-brachial index, SBP systolic blood pressure, DBP diastolic blood pressure, LVEF left ventricular ejection fraction, VHD valvular heart disease

This study was designed as a retrospective analysis conducted at a single tertiary care hospital. The study population consisted of individuals aged 40 to 75 years who visited the cardiovascular center of Boramae Medical Center (Seoul, Republic of Korea) between January 2010 and December 2016 and underwent baPWV measurement during their clinical evaluation. Measurement of baPWV was not part of a mandatory screening program but was performed at the discretion of the attending physician as a component of cardiovascular risk assessment, particularly in patients with known risk factors or those who required further evaluation of vascular aging and arterial stiffness. Thus, the cohort represents a real-world patient population in whom baPWV was used as a clinical adjunct to standard cardiovascular assessment. Initially, a total of 9,975 subjects were screened. To ensure the reliability of baPWV results, the following cases were excluded from the analysis: (1) ankle-brachial index < 0.9 or > 1.4, (2) systolic blood pressure ≥ 180 mmHg or diastolic blood pressure ≥ 110 mmHg, (3) uncontrolled arrhythmia, defined as the presence of atrial fibrillation, atrial flutter with variable conduction, frequent premature beats, or other irregular cardiac rhythms that could interfere with accurate baPWV measurement, (4) impaired left ventricular systolic function with left ventricular ejection fraction < 40%, (5) moderate or greater valvular heart disease, (6) presence of pericardial effusion, defined as moderate or greater effusion on echocardiography or clinically significant effusion that could affect hemodynamic status or vascular measurements (small, physiologic pericardial effusions were not considered exclusion criteria), and (7) congenital heart disease. After these exclusions, 9521 subjects were included in the final analysis. The study enrollment process is illustrated in Fig. 1. The study protocol was reviewed and approved by the institutional review board of Boramae Medical Center (approval number, 20-2025-16) and the requirement for informed consent was waived due to the retrospective nature of the study.

### Data collection of clinical parameters

Body mass index (BMI) was calculated as body weight (kg) divided by the square of height (m²). Hypertension was defined as a prior diagnosis by a physician, the use of antihypertensive medications, or a systolic blood pressure ≥ 140 mmHg and/or diastolic blood pressure ≥ 90 mmHg. Diabetes mellitus was defined as a prior diagnosis by a physician, the use of antidiabetic medications, fasting plasma glucose ≥ 126 mg/dL, or glycated hemoglobin (HbA1c) ≥ 6.5%. A history of coronary artery disease (CAD) included prior myocardial infarction or coronary revascularization. After an overnight fast of approximately 12 h, venous blood samples were collected from the antecubital vein, and laboratory measurements included white blood cell count, hemoglobin, glucose, HbA1c, creatinine, total cholesterol, low-density lipoprotein cholesterol, high-density lipoprotein cholesterol, triglycerides, and C-reactive protein. The estimated glomerular filtration rate (eGFR) was calculated using the Modification of Diet in Renal Disease (MDRD) equation [12]. Laboratory tests were also performed on the same day or during the same clinical visit as baPWV measurement in most participants. Information on concomitant cardiovascular medications was also obtained, including calcium channel blockers, renin–angiotensin system (RAS) blockers, *β*-blockers, and statins.

### baPWV measurement

Arterial stiffness was assessed using baPWV. The detailed methodology for baPWV measurement was described previously [13, 14]. In brief, baPWV was measured with a VP-1000 device (Omron Colin, Komaki, Japan). All examinations were performed in a quiet, temperature-controlled room. Participants rested in the supine position before the procedure to minimize hemodynamic fluctuations. On the day of testing, alcohol, smoking, and caffeine-containing beverages were prohibited, whereas routine medications were continued. Pneumatic cuffs were placed on both arms and ankles, and pulse waves were recorded simultaneously. The device automatically calculated pulse transit time between the brachial and ankle sites, and travel distance was estimated based on body height. baPWV was then calculated as the distance divided by the transit time. All measurements were performed by an experienced technician using a validated automated device. For participants with bilateral values, the mean baPWV was used for analysis. Blood pressure was measured concurrently, and recordings were repeated if artifacts or arrhythmias were observed. In our laboratory, the intra-observer coefficient of variation, determined from 50 subjects, was approximately 5% [15].

### ePWV calculation

ePWV was derived using a previously validated formula [6, 9]. Systolic blood pressure (SBP) and diastolic blood pressure (DBP) were measured at the time of baPWV assessment using an automated oscillometric device after adequate rest. The same blood pressure values were used to calculate mean arterial pressure (MAP) and derive ePWV. MAP was calculated as DBP + 0.4 × (SBP – DBP). The ePWV value was then estimated based on patient age and MAP according to the following regression equation: ePWV (m/s) = 9.587 - (0.402 × age) + (4.560 × 0.001 × age^2^) - (2.621 × 0.00001 × age^2^ × MAP) + (3.176 × 0.001 × age × MAP) - (1.832 × 0.01 × MAP) [6].

### Clinical outcome assessment

The primary endpoint of this study was major adverse cardiovascular events (MACE), defined as a composite of cardiac death, non-fatal myocardial infarction, coronary revascularization, and non-fatal ischemic stroke. Cardiac death was defined as death caused by acute coronary syndrome, heart failure, or ventricular arrhythmia; unexplained sudden death was also considered cardiac death. Acute myocardial infarction was diagnosed based on the presence of chest pain, electrocardiographic changes, elevated cardiac enzymes, and/or supportive coronary angiographic findings. Coronary revascularization included both percutaneous coronary intervention and coronary artery bypass graft surgery. Ischemic stroke was defined as the sudden onset of neurological deficits accompanied by a corresponding ischemic lesion on brain imaging.

### Statistical analysis

Numbers are presented as mean ± standard deviation or as n (%). Differences in clinical parameters between subjects with and without MACE were assessed using Student’s t-test for continuous variables and the chi-square test for categorical variables. Differences in MACE incidence between two groups or among three groups were also analyzed using the chi-square test. For grouping and risk stratification based on baPWV, a cutoff value of 18 m/s was used (high risk, baPWV > = 18 m/s), whereas for ePWV, a cutoff of 10 m/s was applied (high risk, ePWV > = 10 m/s) [16]. Independent associations between PWV and MACE were evaluated using multivariable Cox regression analysis. In this model, the following clinical covariates were adjusted for as potential confounders: age, sex, body mass index, hypertension, diabetes mellitus, cigarette smoking, CAD, use of *β*-blockers, RAS blockers, and statins. To compare model discrimination, we calculated Harrell’s C-index for multivariable Cox proportional hazards models that included identical covariates plus either baPWV or ePWV. Linear predictors from each model were used to estimate the C-index, and paired comparisons were performed on the same individuals. We obtained 95% confidence intervals (CI) and p values using bootstrap resampling with 1000 replicates and a paired test implemented in R (survcomp). Comparisons were conducted for models using dichotomized cutoffs and for models using tertile stratification, with complete-case analysis to ensure identical samples across paired tests. Kaplan–Meier survival curves were constructed to illustrate the prognostic significance of PWV, and the log-rank test was applied to compare event-free survival between groups. All statistical tests were two-tailed, and a *P*-value < 0.05 was considered statistically significant. Statistical analyses were performed using SPSS version 29 (IBM Corp., Armonk, NY, USA) and R version 4.5.1 (R Foundation for Statistical Computing, Vienna, Austria).

## Results

**Fig. 2:**
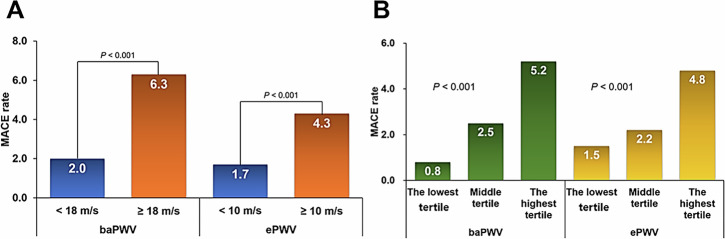
MACE rates by baPWV and ePWV categories. **A** MACE rates according to dichotomized baPWV (cut-off 18 m/s) and ePWV (cut-off 10 m/s). **B** MACE rates according to tertiles of baPWV and ePWV. Tertile ranges were 7.98–14.19 m/s, 14.20–16.46 m/s, and 16.47–39.85 m/s for baPWV, and 4.12–8.92 m/s, 8.93–10.46 m/s, and 10.47–14.63 m/s for ePWV. MACE major adverse cardiovascular event, baPWV brachial-ankle pulse wave velocity, ePWV estimated pulse wave velocity

Among the 9521 study participants, 271 MACE (2.8%) occurred during a median follow-up of 3.77 years (interquartile range, 1.54 – 5.45 years). These included 24 cardiac deaths (0.3%), 31 non-fatal myocardial infarctions (0.3%), 178 coronary revascularizations (1.9%), and 74 non-fatal ischemic strokes (0.8%). In addition, 114 all-cause deaths (1.2%) were observed during follow-up (Table 1). Baseline clinical characteristics of study participants are demonstrated in Table 2. Participants who experienced MACE were older (62.9 ± 8.5 *vs*. 60.2 ± 8.8 years, *P* < 0.001) and more frequently male (65.8% *vs*. 57.8%, *P* = 0.009) compared with those without MACE. BMI and blood pressure values were similar between the two groups. However, participants with MACE had a higher prevalence of cardiovascular risk factors, including hypertension, diabetes mellitus, cigarette smoking, and a prior history of CAD (*P* < 0.05 for each). In laboratory findings, higher levels of white blood cell count, fasting glucose, HbA1c, and C-reactive protein, along with lower levels of hemoglobin, eGFR, total cholesterol, and high-density lipoprotein cholesterol, were observed in participants with MACE. Moreover, cardioprotective medications, such as RAS blockers, *β*-blockers, and statins, were prescribed more frequently in participants with MACE than in those without MACE. The differences in MACE incidences according to PWV values are shown in Fig. 2. Participants with baPWV ≥ 18 m/s had a higher incidence of MACE compared with those with baPWV < 18 m/s (6.3% *vs*. 2.0%; *P* < 0.001). Similarly, individuals with ePWV ≥ 10 m/s experienced significantly more MACEs than those with ePWV < 10 m/s (4.3% *vs*. 1.7%; *P* < 0.001). When patients were stratified into tertiles according to baPWV, the incidence of MACE increased proportionally from the lowest to the highest tertile (from 0.8% to 5.2%; *P* < 0.001), and the same trend was observed for ePWV (from 1.5% to 4.8%; *P* < 0.001).

**Table. 1:** Incidence of clinical events among study participants

Clinical event	Incidence, n (%)
All-cause death	114 (1.2)
Cardiac death	24 (0.3)
Non-fatal acute myocardial infarction	31 (0.3)
Coronary revascularization	178 (1.9)
Ischemic stroke	74 (0.8)
MACE*	271 (2.8)

MACE* includes cardiac death, non-fatal acute myocardial infarction, coronary revascularization, and ischemic stroke. MACE, major adverse cardiovascular event

**Table. 2:** Baseline clinical characteristics of study participants

Characteristic	MACE (+) (n = 271)	MACE (-) (n = 9,250)	*P*
Age, years	62.9 ± 8.5	60.2 ± 8.8	< 0.001
Male sex	178 (65.8)	5435 (57.8)	0.009
BMI, kg/m^2^	25.0 ± 3.1	24.9 ± 3.2	0.213
Systolic blood pressure, mmHg	129 ± 18	128 ± 16	0.319
Diastolic blood pressure, mmHg	76.5 ± 10.4	77.5 ± 10.3	0.059
Heart rate, beats per minute	66.6 ± 11.6	69.5 ± 12.0	0.401
*Cardiovascular risk factors*			
Hypertension	160 (59.0)	4132 (44.7)	<0.001
Diabetes mellitus	99 (36.5)	2146 (23.2)	<0.001
Current smoking	196 (72.3)	75 (27.7)	<0.001
Previous CAD	136 (50.2)	2244 (24.3)	<0.001
Previous stroke	2 (0.7)	50 (0.5)	0.438
*Laboratory findings*			
WBC, per microliter	7885 ± 4442	7142 ± 3033	0.007
Hemoglobin, g/dL	13.3 ± 1.8	13.6 ± 1.7	0.002
Fasting glucose, mg/dL	129 ± 52	120 ± 39	0.039
Glycated hemoglobin, %	6.65 ± 1.19	6.38 ± 1.13	0.033
Estimated GFR, mL/min/1.73m^2^	81.3 ± 25.9	87.2 ± 24.4	<0.001
Total cholesterol, mg/dL	161 ± 42	166 ± 41	0.035
LDL cholesterol, mg/dL	95.4 ± 40.8	98.3 ± 36.6	0.224
HDL cholesterol, mg/dL	45.6 ± 13.5	48.8 ± 12.9	<0.001
Triglycerides, mg/dL	143 ± 101	135 ± 97	0.186
C-reactive protein, mg/dL	2.80 ± 6.81	1.34 ± 4.45	<0.001
*Cardiovascular medications*			
Calcium channel blockers	33 (12.2)	1512 (16.3)	0.067
RAS blockers	133 (49.1)	3144 (34.0)	<0.001
*β-*blockers	110 (40.6)	2197 (23.8)	<0.001
Statins	217 (80.1)	5103 (55.2)	<0.001

Numbers are expressed as mean ± standard deviation or n (%). *MACE* major adverse cardiovascular event, *BMI* body mass index, *CAD* coronary artery disease, *WBC* white blood cell count, *GFR* glomerular filtration rate, *LDL* low-density lipoprotein, *HDL* high-density lipoprotein, *RAS* renin-angiotensin system

In multivariable Cox regression analyses, increased arterial stiffness was significantly associated with a higher risk of MACEs (Table 3). Participants with baPWV ≥ 18 m/s had a 2.78-fold greater risk of experiencing MACEs compared with those with baPWV < 18 m/s (hazard ratio [HR], 2.78; 95% CI, 2.13–3.64; *P* < 0.001). When stratified by baPWV tertiles, the risk of MACEs increased in a stepwise fashion, with hazard ratios of 2.94 (95% CI, 1.88–4.61; *P* < 0.001) in the middle tertile and 5.83 (95% CI, 3.77–9.02; *P* < 0.001) in the highest tertile compared with the lowest tertile. Similarly, ePWV ≥ 10 m/s was independently associated with a 2.46-fold higher risk of MACEs compared with ePWV < 10 m/s (HR, 2.46, 95% CI, 1.79–3.39; *P* < 0.001). In tertile analysis, individuals in the highest ePWV tertile (10.47–14.63 m/s) had a significantly elevated risk of MACEs (HR, 3.50; 95% CI, 2.32–5.26; *P* < 0.001), whereas the association in the middle tertile (8.93 – 10.46 m/s) did not reach statistical significance (HR, 1.44; 95% CI, 0.99–2.11; *P* = 0.056). These findings remained robust after adjusting for potential confounding variables, including age, sex, body mass index, hypertension, diabetes mellitus, cigarette smoking, coronary artery disease, and the use of *β*-blockers, renin–angiotensin system blockers, and statins. In multivariable Cox proportional hazards models with identical covariates, the C-index was 0.736 for baPWV and 0.728 for ePWV using dichotomized cutoffs (*P* = 0.323). With tertile stratification, the C-index was 0.755 for baPWV and 0.729 for ePWV (*P* = 0.033), indicating superior discrimination for baPWV.

**Table. 3:** Multivariable-adjusted risk of MACEs according to arterial stiffness indices

Index for arterial stiffness	HR (95% CI)	*P*
baPWV ≥ 18 m/s	2.78 (2.13–3.64)	< 0.001
baPWV tertile		
The lowest tertile (7.98–14.19 m/s)	1 (reference)	–
Middle tertile (14.20–16.46 m/s)	2.94 (1.88–4.61)	< 0.001
The highest tertile (16.47–39.85 m/s)	5.83 (3.77–9.02)	< 0.001
ePWV ≥ 10 m/s	2.46 (1.79–3.39)	< 0.001
ePWV tertile		
The lowest tertile (4.12–8.92 m/s)	1 (reference)	–
Middle tertile (8.93–10.46 m/s)	1.44 (0.99–2.11)	0.056
The highest tertile (10.47–14.63 m/s)	3.50 (2.32–5.26)	< 0.001

Separate multivariable models were constructed for each arterial stiffness index. The following clinical covariates were controlled as potential confounders during multivariable analyses: age, sex, body mass index, hypertension, diabetes mellitus, cigarette smoking, coronary artery disease, beta-blockers, renin-angiotensin system blockers, and statins. *MACE* major adverse cardiovascular event, *HR* hazard ratio, *CI* confidence interval, *baPWV* brachial-ankle pulse wave velocity, *ePWV* estimated pulse wave velocity

**Fig. 3:**
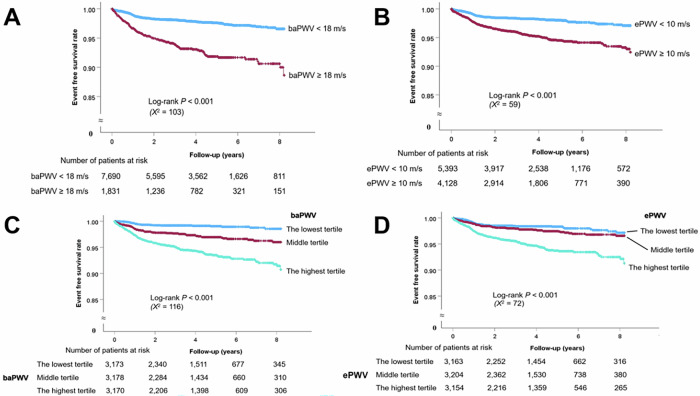
Kaplan–Meier curves for event-free survival by baPWV and ePWV categories. Event-free survival rates according to dichotomized baPWV (cut-off: 18 m/s) (**A**) and ePWV (cut-off: 10 m/s) (**B**), and by tertiles of baPWV (**C**) and ePWV (**D**). baPWV, brachial-ankle pulse wave velocity; ePWV, estimated pulse wave velocity

Kaplan–Meier survival curve analysis was used to visualize the impact of PWV values on MACE-free survival (Fig. 3). The Kaplan–Meier curves demonstrated a significant difference in MACE-free survival between participants with baPWV ≥ 18 m/s and those with baPWV < 18 m/s (log-rank *P* < 0.001). Similarly, individuals with ePWV ≥ 10 m/s had significantly lower MACE-free survival compared with those with ePWV < 10 m/s (log-rank *P* < 0.001), although the degree of separation between the curves was less pronounced than that observed for baPWV (*χ*² = 103 *vs*. 59). In tertile analyses, both baPWV and ePWV showed progressively worse outcomes with increasing tertiles; however, the discriminative power was greater for baPWV than for ePWV (*χ*² = 116 *vs*. 72).

## Discussion

We showed that in adults aged 40–75 undergoing cardiovascular evaluation, both baPWV and ePWV were associated with incident MACE over a median follow-up of 3.77 years. Higher arterial stiffness, as measured by either method, remained independently associated with increased MACE risk after adjustment for clinical covariates. Although baPWV demonstrated stronger predictive performance than ePWV, ePWV still provided significant prognostic information. Because ePWV does not require dedicated measurement equipment, when baPWV is not feasible, these results suggest that ePWV can be used for risk stratification. Our findings extend previous studies by providing a head-to-head comparison of baPWV and ePWV in a large, real-world clinical cohort and by evaluating their relative prognostic performance across multiple risk-stratification approaches.

### Prognostic value of baPWV

The utility of baPWV as a prognostic tool lies in its clinical relevance and operational simplicity, which make large-scale cardiovascular risk stratification feasible in everyday practice and epidemiologic studies [2, 16]. Developed later than carotid-femoral PWV (cfPWV) and popularized in Japan, baPWV uses simple oscillometric cuffs on the arms and ankles to estimate pulse transit time and derive a stiffness index that reflects both central and peripheral arterial beds [17]. This approach minimizes operator dependence, shortens measurement time, and allows large-scale screening in outpatient clinics, health check-up centers, and epidemiologic cohorts. Across numerous studies, particularly in Asian populations, higher baPWV consistently tracks with an adverse cardiometabolic profile, including older age, hypertension, diabetes, chronic kidney disease, and smoking [18]. But critically, it also predicts hard outcomes independent of these covariates. Event analyses repeatedly show that individuals with higher baPWV experience more myocardial infarction, stroke, coronary revascularization, heart failure, and all-cause mortality [3–5, 19, 20]. Also, baPWV adds incremental information to traditional risk scores, standard laboratory markers and imaging studies, improving discrimination and reclassification for composite atherosclerotic events. In our study, which included patients with a moderate- or high-risk cardiovascular profile, baPWV was a strong predictor of incident MACE, consistent with previous reports.

### Prognostic value of ePWV

ePWV is an estimate of arterial stiffness derived from simple clinical inputs, such as age and blood pressure, making it readily available in routine practice and in large datasets without dedicated equipment [6]. Across diverse cohorts, higher ePWV is consistently associated with adverse cardiovascular phenotypes and with a greater incidence of hard outcomes, including all-cause death, cardiovascular death, myocardial infarction, stroke, coronary revascularization, and heart failure [7–9, 21–24]. These associations generally persist after adjustment for conventional risk factors and baseline cardiovascular disease, indicating that ePWV captures additional risk information beyond standard clinical profiles. Because ePWV can be calculated from a single visit and embedded in electronic health records, it enables broad risk screening and longitudinal surveillance at minimal cost, and it is especially useful when baPWV or cfPWV cannot be measured. In our study as well, ePWV demonstrated clear utility for predicting MACE. In settings where device-based stiffness testing is not feasible, ePWV provides actionable stratification that can guide the intensity of prevention and monitoring.

### Comparison of the prognostic values between baPWV and ePWV

A few studies have directly compared baPWV and ePWV and demonstrated that both indices independently predict cardiovascular outcomes and mortality [10, 11]. For example, Hsu et al. [10] reported that both baPWV and ePWV were independent predictors of cardiovascular and all-cause mortality, with ePWV providing superior incremental predictive value for cardiovascular mortality. In our cohort, we directly compared the prognostic performance of baPWV with that of ePWV for predicting subsequent MACE. Both indices were independently associated with adverse outcomes after multivariable adjustment, but baPWV demonstrated consistently stronger discrimination and risk stratification than ePWV. This finding may be biologically plausible. baPWV is a cuff-based physiologic measurement that captures pulse-wave transit across central–peripheral segments and therefore integrates arterial wall properties beyond age and blood pressure, whereas ePWV is a model-derived surrogate primarily informed by those two inputs. As a result, baPWV appears more sensitive to heterogeneity in vascular aging, subclinical atherosclerosis, and cumulative exposure to cardiometabolic stressors, yielding clearer separation of event risk across categories. These findings support a practical hierarchy for clinical use. When resources and workflow allow, baPWV should be preferred for risk stratification, particularly in patients with intermediate baseline risk, multiple comorbidities, or when treatment intensification is being considered. However, ePWV retains clear prognostic utility and offers important advantages in settings where device-based testing is not feasible, such as primary care, large administrative datasets, historical cohorts lacking stiffness measurements, or follow-up venues requiring rapid, low-cost screening. In such settings, ePWV can flag higher-risk patients who may benefit from closer monitoring, optimization of blood pressure and lipids, or confirmatory stiffness testing with baPWV. Taken together, our results indicate that baPWV provides the stronger primary predictor, but ePWV remains a valuable, widely applicable alternative that meaningfully refines cardiovascular risk assessment when baPWV cannot be obtained.

### Study limitations

This study has limitations. It is a retrospective, single-center analysis in which baPWV testing was ordered at the physician's discretion, which introduces selection bias. Additionally, not all participants underwent echocardiographic evaluation, as echocardiography was performed at the discretion of the treating physician in routine clinical practice. This may have led to incomplete identification of structural heart disease and may introduce selection bias. Although models adjusted for many covariates, residual confounding is likely because physical activity, socioeconomic status, and medication adherence were not available. We used baPWV rather than cfPWV, considered the noninvasive gold standard for arterial stiffness, which may limit comparability with other studies. The composite outcome included coronary revascularization, a component influenced by practice patterns, and the low numbers of cardiac death and myocardial infarction reduced power for component-specific analyses. Finally, our data were obtained from Korean adults aged 40–75 years, so extrapolation to other age ranges, care settings, or ethnic groups should be made with caution.

### Perspective of Asia

In Asia, baPWV is widely used in routine clinical practice and large-scale screening programs due to its simplicity and reproducibility, whereas carotid-femoral PWV is more commonly used in Western settings [2]. In this real-world Asian cohort, both baPWV and ePWV were independently associated with cardiovascular risk, with baPWV demonstrating superior risk discrimination. Given the heterogeneity of healthcare systems and resource availability across Asia, ePWV may serve as a practical alternative when device-based measurements are not feasible. These findings support a complementary and pragmatic approach to arterial stiffness assessment in Asian clinical practice.

## Conclusions

Both baPWV and ePWV independently predict MACE beyond traditional risk factors in adults aged 40–75 years with moderate to high cardiovascular risk. baPWV provided stronger risk discrimination than ePWV in this population. ePWV remains a practical option when device-based testing is unavailable. Future multicenter studies are needed to validate standardized thresholds and establish the incremental clinical utility of ePWV.
